# Mental fatigue induced by prolonged self-regulation does not exacerbate central fatigue during subsequent whole-body endurance exercise

**DOI:** 10.3389/fnhum.2015.00067

**Published:** 2015-02-25

**Authors:** Benjamin Pageaux, Samuele M. Marcora, Vianney Rozand, Romuald Lepers

**Affiliations:** ^1^Endurance Research Group, School of Sport & Exercise Sciences, University of Kent at MedwayChatham Maritime, UK; ^2^Laboratoire INSERM U1093, Faculté des Sciences du Sports – UFR Staps, Université de BourgogneDijon, France

**Keywords:** muscle fatigue, mental exertion, neuromuscular fatigue, perceived exertion, perception of effort, sense of effort, Stroop task, response inhibition

## Abstract

It has been shown that the mental fatigue induced by prolonged self-regulation increases perception of effort and reduces performance during subsequent endurance exercise. However, the physiological mechanisms underlying these negative effects of mental fatigue are unclear. The primary aim of this study was to test the hypothesis that mental fatigue exacerbates central fatigue induced by whole-body endurance exercise. Twelve subjects performed 30 min of either an incongruent Stroop task to induce a condition of mental fatigue or a congruent Stroop task (control condition) in a random and counterbalanced order. Both cognitive tasks (CTs) were followed by a whole-body endurance task (ET) consisting of 6 min of cycling exercise at 80% of peak power output measured during a preliminary incremental test. Neuromuscular function of the knee extensors was assessed before and after CT, and after ET. Rating of perceived exertion (RPE) was measured during ET. Both CTs did not induce any decrease in maximal voluntary contraction (MVC) torque (*p* = 0.194). During ET, mentally fatigued subjects reported higher RPE (mental fatigue 13.9 ± 3.0, control 13.3 ± 3.2, *p* = 0.044). ET induced a similar decrease in MVC torque (mental fatigue –17 ± 15%, control –15 ± 11%, *p* = 0.001), maximal voluntary activation level (mental fatigue –6 ± 9%, control –6 ± 7%, *p* = 0.013) and resting twitch (mental fatigue –30 ± 14%, control –32 ± 10%, *p* < 0.001) in both conditions. These findings reject our hypothesis and confirm previous findings that mental fatigue does not reduce the capacity of the central nervous system to recruit the working muscles. The negative effect of mental fatigue on perception of effort does not reflect a greater development of either central or peripheral fatigue. Consequently, mentally fatigued subjects are still able to perform maximal exercise, but they are experiencing an altered performance during submaximal exercise due to higher-than-normal perception of effort.

## INTRODUCTION

Self-regulation is the modulation of thought, affect, behavior, or attention via deliberate or automated use of cognitive control mechanisms (Karoly, 1993) such as response inhibition (Ridderinkhof et al., 2004). Although the effect size may be exaggerated because of publication bias (Carter and McCullough, 2013), several psychological studies have shown that few minutes of engagement with cognitive tasks (CTs) requiring self-regulation (e.g., incongruent Stroop task) can lead to impaired performance in subsequent tasks also requiring self-regulation, including physical tasks like sustained handgrip exercise (Hagger et al., 2010). This phenomenon is often referred to as self-regulatory or ego depletion because the prominent explanation is that self-regulation relies on a limited resource that, when depleted, leads to impaired self-regulation (Muraven and Baumeister, 2000).

In the context of whole-body exercise physiology, we and others found that prolonged (30–90 min) engagement with CTs requiring self-regulation impairs endurance performance during subsequent running or cycling exercise (Marcora et al., 2009; MacMahon et al., 2014; Pageaux et al., 2014). In this context, the prominent explanation for impaired endurance performance is that prolonged engagement with CTs requiring self-regulation induces a subjective state of mental fatigue characterized by feelings of tiredness/lack of energy at rest and/or higher-than-normal perception of effort during subsequent whole-body endurance exercise. In these studies, no negative effects of mental fatigue were found on the physiological systems (cardiorespiratory and metabolic) supporting whole-body endurance exercise. As motivation related to the endurance tasks (ETs) was also unaffected, the authors ascribed the observed impairment in endurance performance to the higher-than-normal perception of effort experienced by mentally fatigued subjects. Indeed, as stated by the psychobiological model of endurance performance (Marcora et al., 2008; Marcora and Staiano, 2010), exhaustion is not caused by muscle fatigue (i.e., by the inability to produce the force/power required by the ET despite a maximal voluntary effort), but is caused by the conscious decision to disengage from the ET. In highly motivated subjects, this effort-based decision is taken when they perceive their effort to be maximal and continuation of the ET seems impossible. During time to exhaustion tests at a fixed workload, higher-than-normal perception of effort means that mentally fatigued subjects reach their maximal perceived effort and disengage from the ET prematurely (Marcora et al., 2009; Pageaux et al., 2013). During self-paced time trials (Pageaux, 2014), the psychobiological model correctly predicts that mentally fatigued subjects consciously reduce the power output/speed in order to compensate for the higher-than-normal perception of effort and, thus, avoid premature exhaustion (Marcora, 2010a; Pageaux, 2014).

Although the psychobiological model seems to provide a valid explanation for the negative effects of mental fatigue on endurance performance, at present we cannot totally exclude the possibility that the negative effects of mental fatigue on endurance performance may be mediated, at least in part, by the central component of muscle fatigue: central fatigue [operationally defined as an exercise-induced decrease in maximal voluntary activation level (VAL); Gandevia, 2001]. This is relevant because, similarly to mental fatigue, muscle fatigue can also increase perception of effort and reduce performance during ETs (Marcora et al., 2008; de Morree and Marcora, 2013). Pageaux et al. (2013) recently assessed neuromuscular function of the knee extensors before and after a prolonged CT requiring self-regulation (90-min AX continuous performance task), and after a subsequent ET (submaximal isometric knee extensor exercise until exhaustion). The authors found that mental fatigue did not decrease VAL during maximal voluntary contraction (MVC) of the knee extensors before the ET, and that mental fatigue did not exacerbate central fatigue induced by the subsequent ET. Although these findings suggest that mental fatigue does not reduce the capacity of the central nervous system (CNS) to recruit the working muscles, it has to be noticed that neuromuscular function was not assessed for the same duration of exercise between conditions. Because mental fatigue reduced time to exhaustion, exercise duration was significantly different between conditions and it is possible that mental fatigue increased the rate of central fatigue development compared to the control condition. Furthermore, it is well-known that muscle fatigue is task specific (Bigland-Ritchie et al., 1995) and that both neural control of movement and systemic stress differ between single-joint and whole-body exercise (Sidhu et al., 2013). Of particular interest is the fact whole-body endurance exercise is known to induce homeostatic disturbances within the CNS that may influence central fatigue (for review see Nybo and Secher, 2004). It is therefore possible that mental fatigue can interact with these processes leading to greater central fatigue when neuromuscular function is measured after the same duration of whole-body endurance exercise.

The primary aim of this study was to test the hypothesis that mental fatigue induced by a prolonged CT requiring strong response inhibition (30-min incongruent Stroop task) exacerbates central fatigue during subsequent whole-body endurance exercise. As perception of effort can be increased by muscle fatigue (Marcora et al., 2008; de Morree et al., 2012; de Morree and Marcora, 2013), we examined both central fatigue and peripheral fatigue (i.e., fatigue produced by changes at or distal to the neuromuscular junction; Gandevia, 2001) before and after the incongruent Stroop task. Neuromuscular function was also examined after a whole-body ET consisting of 6 min of high-intensity cycling exercise in order to control for the confounding effects of exercise duration.

## MATERIALS AND METHODS

### SUBJECTS AND ETHICAL APPROVAL

Twelve physically active male adults (mean ± SD; age: 25 ± 4 years, height: 182 ± 5 cm, weight: 77 ± 11 kg) volunteered to participate in this study. None of the subjects had any known mental or somatic disorder. “Active” was defined as taking part in moderate to high intensity exercise at least twice a week for a minimum of 6 months. Our subjects can be included in the performance level 2 in the classification of subject groups in sport science research (de Pauw et al., 2013). Each subject gave written informed consent prior to the study. Experimental protocol and procedures were approved by the local Ethics Committee of the Faculty of Sport Sciences, University of Burgundy in Dijon. All subjects were given written instructions describing all procedures related to the study but were naive of its aims and hypotheses. At the end of the last visit, subjects were debriefed and asked not to discuss the real aims of the study with other participants. The study conformed to the standards set by the World Medical Association (2013).

### EXPERIMENTAL PROTOCOL

Subjects visited the laboratory on three different occasions. During the first visit, a preliminary incremental test (2 min at 50 W + 50 W increments every 2 min) was performed until exhaustion (defined as a cadence below 60 RPM for more than 5 s despite strong verbal encouragement) on an electromagnetically braked cycle ergometer (Excalibur Sport, Lode, Groningen, The Netherlands) to measure peak power output (303 ± 30 W). The cycle ergometer was set in hyperbolic mode, which allows the power output to be regulated independently of cadence over the range of 30–120 RPM. Before the incremental test the position on the cycle ergometer was adjusted for each subject, and settings were recorded and reproduced at each subsequent visit. Thirty minutes after the incremental test, subjects were familiarized with all experimental procedures.

**FIGURE 1:**
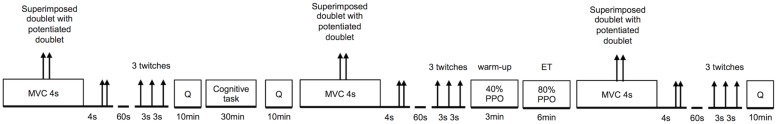
**Graphical overview of the experimental protocol.** Order and timing were the same for each subject and each visit. Q, psychological questionnaires; PPO, peak power output; MVC, maximal voluntary contraction; ET, whole-body endurance task.

During the second and third visit, subjects performed a 30-min CT either involving response inhibition (self-regulation task) or a control task (see *Cognitive Tasks*) in a randomized and counterbalanced order. After the CTs and a short warm up, subjects performed 6 min of high intensity cycling exercise at a fixed workload (see *Whole-Body Endurance Task*). Neuromuscular function of the knee extensors was tested before and after the CTs, and after the whole-body ET (see *Neuromuscular Function Tests*). Mood was assessed before and after the CTs, subjective workload was assessed after the CTs and after the ET. For more details see *Physiological and Psychological Measurements*. An overview of the experimental protocol performed during the second and third visit is presented in **Figure 1**. Heart rate (HR) was recorded continuously throughout the experiment. Each participant completed all three visits over a period of 2 weeks with a minimum of 48 h recovery period between visits. All participants were given instructions to sleep for at least 7 h, refrain from the consumption of alcohol, and not to practice vigorous physical activity the day before each visit. Participants were also instructed not to consume caffeine and nicotine for at least 3 h before testing, and were asked to declare if they had taken any medication or had any acute illness, injury, or infection.

### COGNITIVE TASKS

Both CTs were performed for 30 min, and they are identical to those used by Pageaux et al. (2014) to reduce self-paced endurance running performance. An incongruent Stroop task and a congruent Stroop task were used respectively for the self-regulation task and the control task (Stroop, 1992). A brief description of these CTs can be found below.

#### Self-regulation task

The modified incongruent Stroop task used as self-regulation task consisted of color words (yellow, blue, green, red) printed in a different ink color (either yellow, blue, green, red) presented on a computer screen. Subjects were instructed to press one of four colored buttons on the computer keyboard (yellow, blue, green, red) with the correct response being the button corresponding to the ink color (either yellow, blue, green, red) of the word presented on the computer screen. If however, the ink color was red, the button to be pressed was the button linked to the real meaning of the word, not the ink color (e.g., if the word blue appears in red, the button blue has to be pressed). If the ink color was blue, green or yellow, then the button pressed matched the ink color. The word presented and its ink color were randomly selected by the computer (100% incongruent). Subjects were instructed to respond as quickly and accurately as possible. Feedback (correct or incorrect response, reaction time, and response accuracy so far) was provided on the computer screen after each word. Participants were also informed that points would be awarded for speed and accuracy of their responses, and the score for both CTs would be added to the score for each time trial.

#### Control task

The congruent version of the Stroop color-word task used as control task was similar to the modified incongruent version of the Stroop color-word task. However, all words and their ink color were matched in order to greatly reduce the extent of self-regulation required by the CT. Subjects were familiarized with both CTs during the first visit to the laboratory. Response accuracy (percentage of correct responses) and reaction time were measured to monitor cognitive performance. Data were averaged every 5 min and analyzed oﬄine using the E-Prime software (Psychology Software Tools, Pittsburgh, PA, USA). No filters were applied to trim the reaction time data.

### WHOLE-BODY ENDURANCE TASK

Fifteen minutes after completion of the CT, subjects performed the whole-body ET on an electromagnetically braked cycle ergometer (Excalibur Sport, Lode, Groningen, The Netherlands) set in hyperbolic mode. After a 3-min warm-up cycling at 40% of peak power output (121 ± 12 W), subjects cycled at 80% of peak power output (242 ± 23 W) for 6 min. Cadence was freely chosen between 60 and 100 RPM, and a fan was placed in a standardized position in front of the subject during the entire duration of the task. Feedback on elapsed time, cadence, power output, and HR was not available to the subject. Once the 6 min were elapsed, subjects stopped cycling immediately and were transferred to the isokinetic dynamometer for the assessment of neuromuscular function (see *Neuromuscular Function Tests*). At the end of the warm-up, and at the end of each minute thereafter, rating of perceived exertion (RPE) and cadence were recorded. Subjects were familiarized with the whole-body ET during the first visit to the laboratory.

### NEUROMUSCULAR FUNCTION TESTS

All participants were familiarized with all neuromuscular function tests during their first visit to the laboratory. The neuromuscular function tests performed in this study are identical as those performed by Pageaux et al. (2013).

#### Electrical stimulation

Both single and double (100 Hz frequency) stimulation were used for assessment of neuromuscular function. All central fatigue parameters were obtained within 45 s after completion of the whole-body ET. Transcutaneous electric1ally evoked contractions of the knee extensor muscles were induced by using a high-voltage (maximal voltage 400 V) constant-current stimulator (model DS7 modified, Digitimer, Hertfordshire, UK). A monopolar cathode ball electrode (0.5 cm diameter) pressed into the femoral triangle by the same experimenter during all tests was used to stimulate the femoral nerve. To ensure reliability of measurement, the site of stimulation producing the largest resting twitch amplitude and compound muscle action potential (M-wave) was marked on the skin with permanent marker. The anode was a 50 cm^2^ (10 × 5 cm) rectangular electrode (Compex SA, Ecublens, Switzerland) located on the gluteus maximus opposite to the cathode. The stimulus intensity required to evoke a maximal compound muscle action potential (M_max_) was determined at rest and during submaximal isometric knee extensors contractions (50% MVC) before the experiment on each day. The stimulus duration was 1 ms and the interval of the stimuli in the doublet was 10 ms. Supramaximal intensities ranged from 74 to 140 mA. Timing of stimulation was as follow (see **Figure 1**): (i) MVC (duration of ∼4 s) with superimposed supramaximal paired stimuli (doublet) at 100 Hz and followed (4 s intervals) by paired stimuli at 100 Hz, (ii) 60 s rest and (iii) three single supramaximal stimulations at rest (interspaced by 3 s). Methodology and supramaximal intensities are according to previous studies (e.g., Place et al., 2005; Pageaux et al., 2013).

#### Mechanical recordings

An isokinetic dynamometer (Biodex Medical Systems Inc., Shirley, NY, USA) was used to record the torque signal. The axis of the dynamometer was aligned with the knee axis, and the lever arm was attached to the shank with a strap. Two crossover shoulder harnesses and a belt limited extraneous movement of the upper body. Neuromuscular function tests were performed with a knee angle of 90° of flexion (0° = knee fully extended) and a hip angle of 90°. The following parameters were analyzed from the twitch response (average of 3 single stimulation interspaced by 3 s): peak twitch (Tw), time to peak twitch (contraction time, Ct), average rate of force development (RFD = Tw/Ct), and half-relaxation time. The peak torque of the doublet (potentiated doublet, 5 s after the MVC) was also analyzed. MVC torque was considered as the peak torque attained during the MVC, and guidelines to perform MVCs were respected (Gandevia, 2001). VAL during the MVC was estimated according to the following formula:

$$VAL=(1-\frac{\sup erimposed\,\;\;doublet\,\;amplitude}{potentiated\,\;\;doublet\,\;amplitude})*100$$

Because of technical issue (no potentiated doublet for one subject as the stimulator wire was damaged), VAL and doublets were analyzed only for 11 on 12 subjects. Mechanical signals were digitized on-line at a sampling frequency of 1 kHz using a computer, and stored for analysis with commercially available software (AcqKnowledge 4.1 for MP Systems, Biopac Systems Inc., Goleta, CA, USA).

#### Electromyographic recordings

Electromyogram (EMG) of the vastus lateralis (VL) and rectus femoris (RF) muscles was recorded with pairs of silver chloride circular (recording diameter of 10 mm) surface electrodes (Swaromed, Nessler Medizintechnik, ref 1066, Innsbruck, Austria) with an interelectrode (center-to-center) distance of 20 mm. Low resistance between the two electrodes (<5 kΩ) was obtained by shaving the skin and removing the dirt from the skin using alcohol swabs. The reference electrode was attached to the patella of the right knee. Myoelectrical signals were amplified with a bandwidth frequency ranging from 10 to 500 Hz (gain = 1000 for RF and 500 for VL), digitized on-line at a sampling frequency of 2 kHz using a computer, and stored for analysis with a commercially available software (AcqKnowledge 4.1 for MP Systems, Biopac Systems Inc., Goleta, CA, USA). The root mean square (RMS), a measure of EMG amplitude, was automatically calculated with the software. Peak-to-peak amplitude of the M-waves were analyzed for VL and RF muscles with the average of the three trials used for analysis. EMG amplitude of VL and RF muscles during the MVC was quantified as the RMS for a 0.5 s interval at peak torque (250 ms interval either side of the peak torque). Maximal EMG RMS values for VL and RF muscles were then normalized by the M-wave peak-to-peak amplitude for the respective muscles, in order to obtain the RMS/M-wave ratio. This normalization procedure accounted for peripheral influences such as neuromuscular propagation failure. EMG RMS was calculated for the last 30 s of each minutes during the whole-body ET for both VL and RF. The EMG RMS during the whole-body ET was normalized to the EMG RMS of the last 30 s of the first minute of the whole-body ET.

### PHYSIOLOGICAL AND PSYCHOLOGICAL MEASUREMENTS

All participants were familiarized with all psychological measurements during their first visit to the laboratory. The psychological measurements performed in this study are identical as those performed by Pageaux et al. (2014).

#### Heart rate

Heart rate was recorded continuously during both CTs and the whole-body ET using a HR monitor (Polar RS400, Polar Electro Oy, Kempele, Finland) with an acquisition frequency of 5 sample/s. Data were analyzed oﬄine and averaged for both CTs. During the whole-body ET, HR data were averaged every minute.

#### Perception of effort

During the whole-body ET, perception of effort was measured at the end of the warm-up and every minute thereafter using the 15 points RPE scale (Borg, 1998). Standardized instructions for memory anchoring of the scale were given to each subject before the warm-up. Briefly subjects were asked to rate the conscious sensation of how hard, heavy, and strenuous the physical task was (Marcora, 2010b). For example nine corresponds to a “very light” exercise. For a normal, healthy person it is like walking slowly at his or her own pace for some minutes. Seventeen corresponds to a “very hard” and strenuous exercise. A healthy person can still go on, but he or she really has to push him or herself. It feels very heavy, and the person is very tired.

#### Mood

The Brunel Mood Scale (BRUMS) developed by Terry et al. (2003) was used to quantify current mood (“How do you feel right now?”) before and after the CTs. This questionnaire contains 24 items (e.g., “angry, uncertain, miserable, tired, nervous, energetic”) divided into six subscales: anger, confusion, depression, fatigue, tension, and vigor. The items are answered on a five points scale (0 = not at all, 1 = a little, 2 = moderately, 3 = quite a bit, 4 = extremely), and each subscales, with four relevant items, can achieve a raw score in the range of 0–16. Only scores for the Fatigue and vigor subscales were considered in this study as subjective markers of mental fatigue.

#### Subjective workload

The National Aeronautics and Space Administration Task Load Index (NASA-TLX; Hart and Staveland, 1988) was used to assess subjective workload. The NASA-TLX is composed of six subscales: Mental Demand (How much mental and perceptual activity was required?), Physical Demand (How much physical activity was required?), Temporal Demand (How much time pressure did you feel due to the rate or pace at which the task occurred?), Performance (How much successful do you think you were in accomplishing the goals of the task set by the experimenter?), Effort (How hard did you have to work to accomplish your level of performance?), and Frustration (How much irritating, annoying did you perceive the task?). The participants had to score each of the items on a scale divided into 20 equal intervals anchored by a bipolar descriptor (e.g., High/Low). This score was multiplied by 5, resulting in a final score between 0 and 100 for each of the six subscales. Participants completed the NASA-TLX after the CT and after the whole-body ET.

### STATISTICS

All data are presented as means ± standard deviation (SD) unless stated. Assumptions of statistical tests such as normal distribution and sphericity of data were checked as appropriate. Lower-Bound correction to the degrees of freedom was applied when violations to sphericity were present. Paired *t*-tests were used to assess the effect of condition (mental fatigue vs. control) on HR during both CTs and on NASA-TLX scores after the CTs and after the whole-body ET. Fully repeated measure 2 × 6 ANOVAs were used to test the effects of condition and time on response accuracy and reaction time during the CTs. Fully repeated measure 2 × 2 ANOVAs were used to test the effects of condition and time on mood before and after the CTs. Fully repeated measure 2 × 3 ANOVAs were used to test the effects of condition and time on MVC torque, VAL, M-wave parameters for each muscle, RMS/M-wave ratio, twitch properties, and peak doublet torque before and after the CTs, and after the whole-body ET. Fully repeated measure 2 × 6 ANOVAs were used to test the effects of condition and time on HR, and EMG RMS during the whole-body ET. Fully repeated measure 2 × 7 ANOVA was used to test the effects of condition and time on RPE and cadence during the whole-body ET. Significant main effects of time and significant interactions were followed up with Bonferroni tests as appropriate. Significance was set at 0.05 (2-tailed) for all analyses, which were conducted using the Statistical Package for the Social Sciences, version 20 for Mac OS X (SPSS Inc., Chicago, IL, USA). Cohen’s effects size *d*_z_ and f(V) were calculated with G*Power software (version 3.1.6, Universität Düsseldorf, Germany) and reported.

## RESULTS

### COGNITIVE TASKS

#### Mood

Self-reported fatigue was significantly higher [*p* = 0.009, *f(V)* = 0.957] post-CTs (mental fatigue condition 3.7 ± 3.4, control condition 4.5 ± 3.6) compared to pre-CTs (mental fatigue condition 1.5 ± 2.0, control condition 1.8 ± 1.5). However, neither the main effect of condition [*p* = 0.369, *f(V)* = 0.951] nor the interaction [*p* = 0.401, *f(V)* = 0.264] were significant. Vigor decreased [*p* = 0.009, *f(V)* = 0.283] significantly after the self-regulation task (10.2 ± 3.0 to 8.3 ± 3.9) and the control task (10.6 ± 4.0 to 7.8 ± 4.7) with no significant difference between conditions [interaction *p* = 1.000, *f(V)* = 0.032].

#### Cognitive performance

Response accuracy during CTs did not present any main effect of condition [*p* = 0.070, *f(V)* = 0.605] or time [*p* = 0.236, *f(V)* = 0.378]. Reaction time during both conditions did not change over time [*p* = 0.507, *f(V)* = 0.207] but was significantly longer during the self-regulation task compared to the control task [834 ± 109 vs. 597 ± 80 ms, *p* < 0.001, *f(V)* = 2.500]. Reaction time during the self-regulation task was significantly higher for all subjects.

#### Heart rate

Heart rate was significantly higher (*p* < 0.001, *d*_z_ = 0.577) during the self-regulation task (65.8 ± 9.3 beats/min) compared to the control task (62.0 ± 4.5 beats/min).

#### Subjective workload

**FIGURE 2:**
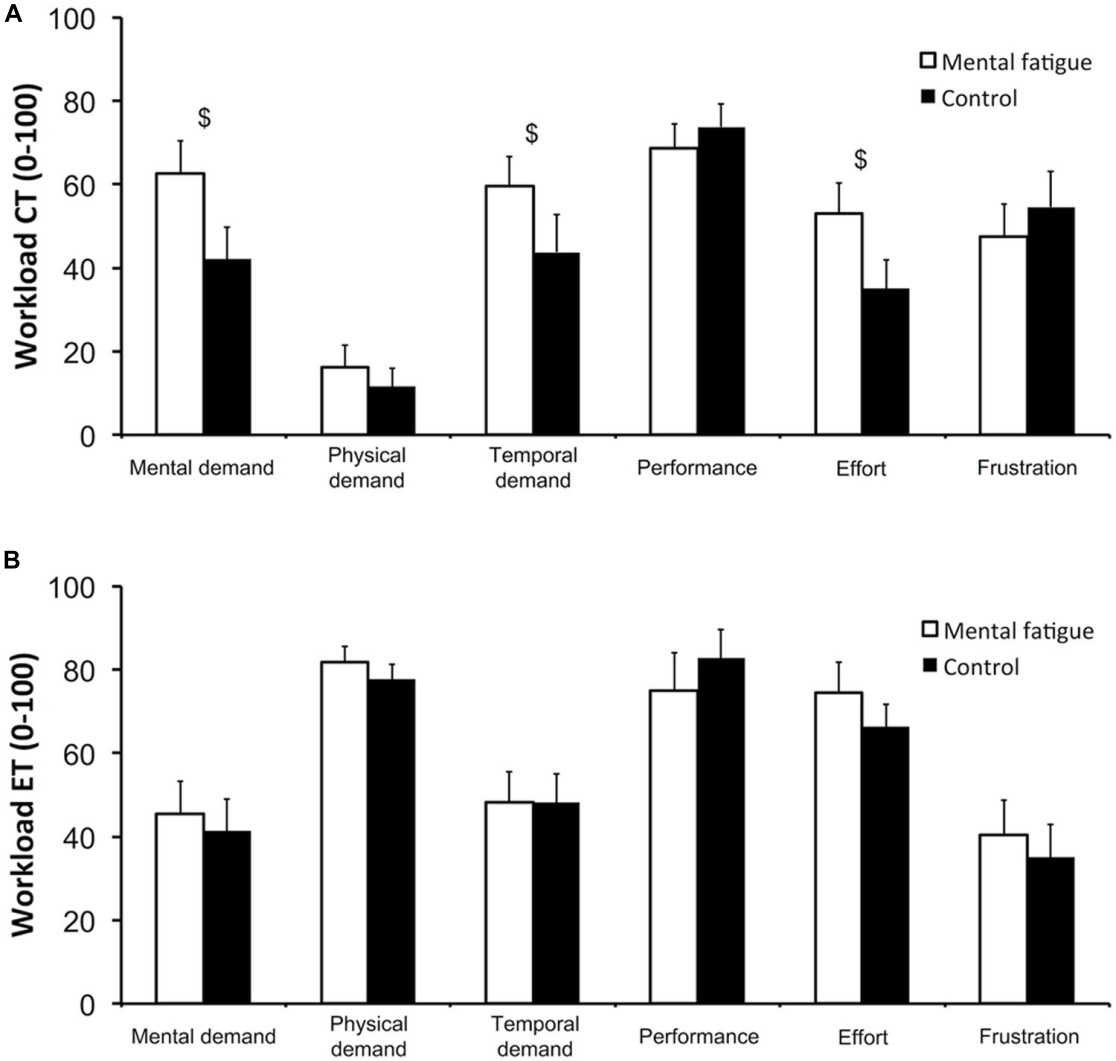
**Subjective workload of the cognitive tasks (CTs, **A**) and of the whole-body endurance task (ET, **B**).** National Aeronautics and Space Administration Task Load Index (NASA-TLX) subscales. $ Significant main effect of condition (*p* < 0.05). Data are presented as mean ± SEM.

Data on all six subscales of the NASA-TLX are presented in **Figure 2**. Following the CTs (**Figure 2A**), subjects rated higher mental demand (*p* = 0.012, *d*_z_ = 0.861), temporal demand (*p* = 0.050, *d*_z_ = 0.626) and effort (*p* = 0.022, *d*_z_ = 0.772) during the self-regulation task (mental fatigue condition) than during the control task (control condition). Physical demand, performance and frustration did not differ significantly between conditions.

### EFFECTS OF MENTAL FATIGUE ON THE PHYSIOLOGICAL AND PSYCHOLOGICAL RESPONSES TO THE SUBSEQUENT WHOLE-BODY ENDURANCE TASK

#### Heart rate

**FIGURE 3:**
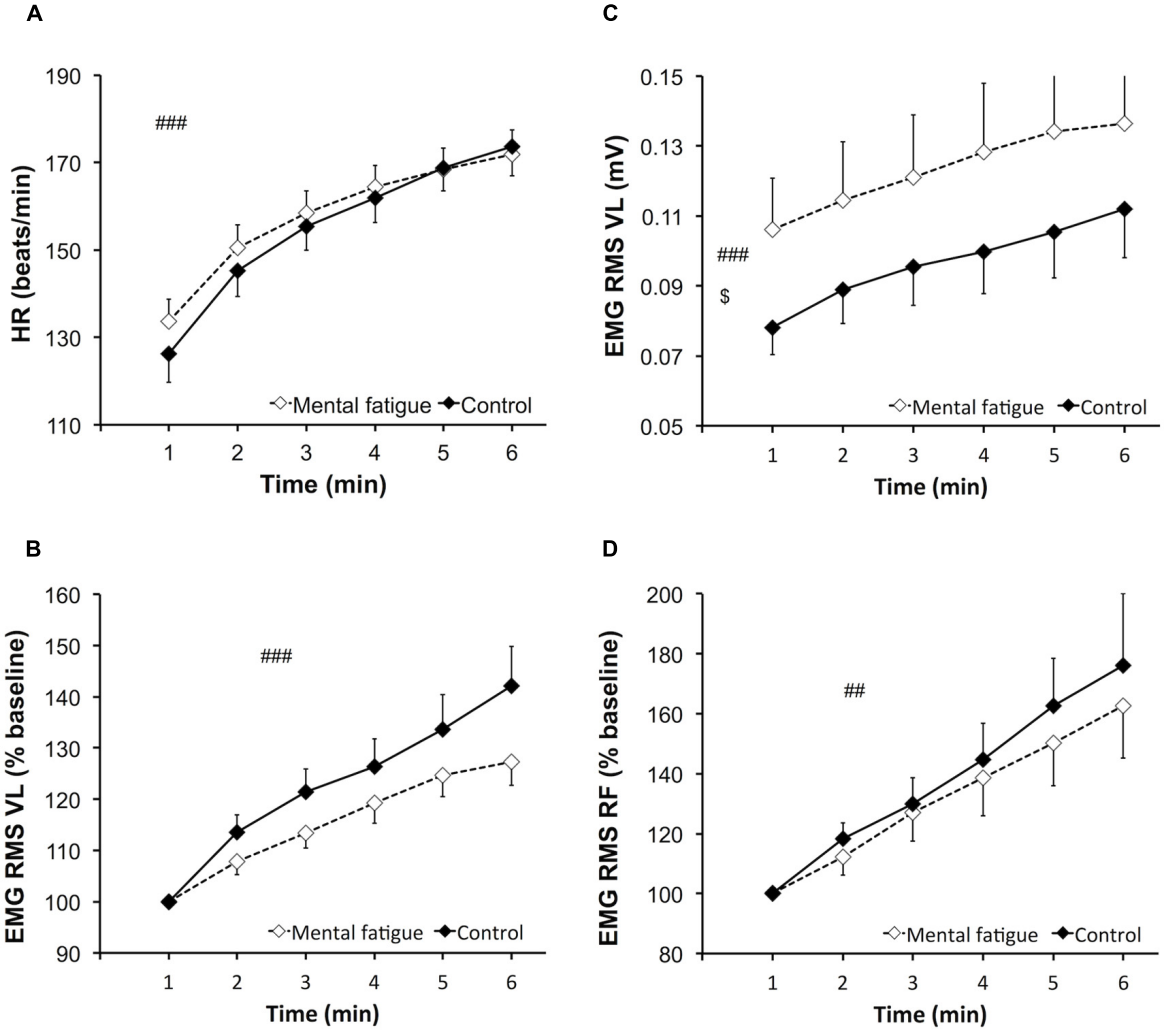
**Effects of mental fatigue on heart rate and electromyogram (EMG) amplitude of the knee extensors during the whole-body endurance task (ET).** Heart rate (HR) during ET **(A)**. EMG root mean square (RMS) for the vastus lateralis (VL) muscle normalized by the first minute of ET (baseline; **B**). EMG RMS of the VL muscle during ET **(C)**. EMG RMS for the rectus femoris (RF) muscle normalized by the first minute of ET (baseline; **D**). $ Significant main effect of condition (*p* < 0.05). ## Significant main effect of time (*p* < 0.01). ### Significant main effect of time (*p* < 0.001). Data are presented as mean ± SEM.

Heart rate (**Figure 3A**) increased significantly over time [*p* < 0.001, *f(V)* = 4.776] but did not differ between conditions [*p* = 0.381, *f(V)* = 0.274].

#### Cadence and EMG amplitude

Cadence (mental fatigue condition 84.4 ± 5.4 RPM, control condition 84.2 ± 6.0 RPM) during the whole-body ET did not present any main effect of condition [*p* = 0.919, *f(V)* = 0.031], time [*p* = 0.175, *f(V)* = 0.418], or interaction [*p* = 0.101, *f(V)* = 0.412].

Electromyogram amplitude data are presented in **Figure 3**. EMG RMS of the VL muscle (**Figure 3C**) increased significantly during the whole-body ET [*p* = 0.002, *f(V)* = 1.25]. EMG RMS of the VL muscle was significantly higher during the mental fatigue condition compared to the control condition [*p* = 0.046, *f(V)* = 0.678]. EMG RMS of the RF muscle increased significantly during the whole-body ET [*p* = 0.002, *f(V)* = 1.305] without any main effect of condition [*p* = 0.610, *f(V)* = 0.167] or interaction [*p* = 0.626, *f(V)* = 0.160]. Time course of EMG RMS for the VL (**Figure 3B**) and RF (**Figure 3D**) muscles did not differ between conditions [VL, *p* = 0.111, *f(V)* = 0.523; RF, *p* = 0.410, *f(V)* = 0.272] and did not present a significant interaction [VL, *p* = 0.091, *f(V)* = 0.557; RF, *p* = 0.384, *f(V)* = 0.289].

#### Perception of effort

**FIGURE 4:**
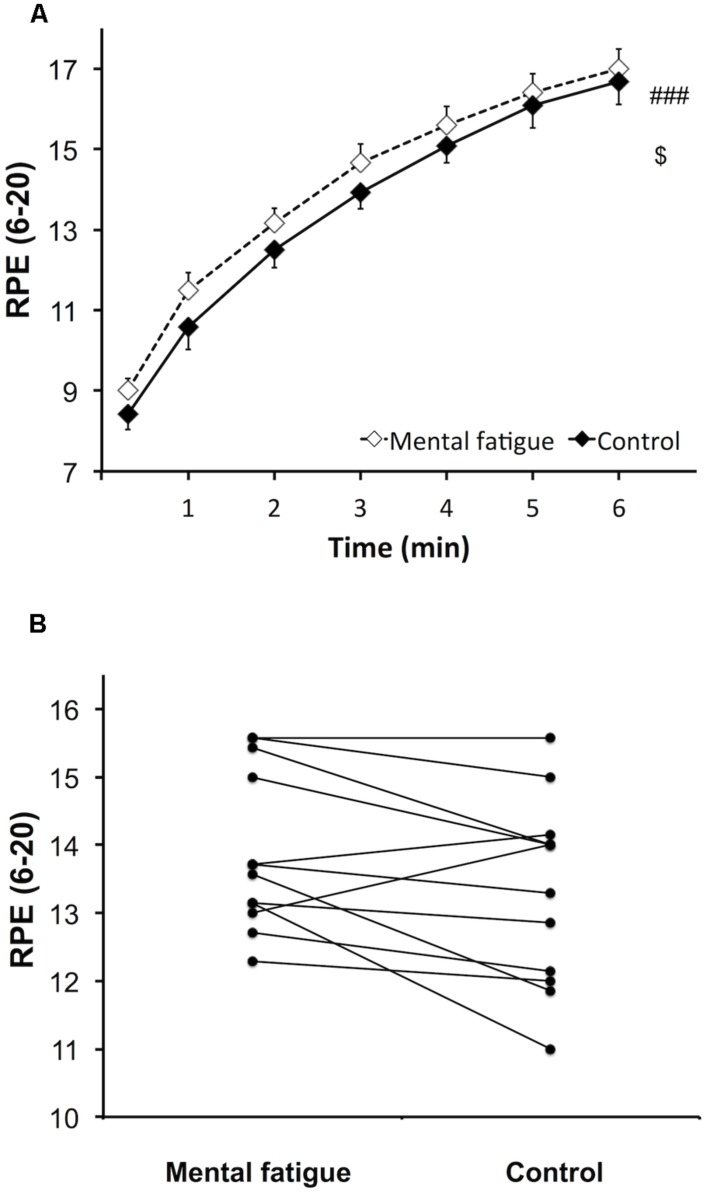
**Effects of mental fatigue on perception of effort during the whole-body ET.** Overall rating of perceived exertion (RPE) during ET **(A)**. Individual effects of condition on the mean overall RPE during ET **(B)**. ### Significant main effect of time (*p* < 0.001). $ Significant main effect of condition (*p* < 0.05). Data are presented as mean ± SEM.

Rating of perceived exertion during the whole-body ET (**Figure 4A**) increased over time following both CTs [*p* < 0.001, *f(V)* = 3.590]. However, subjects rated a higher perceived exertion during the mental fatigue condition compared to the control condition [*p* = 0.044, *f(V)* = 0.680]. No significant interaction was demonstrated [*p* = 0.630, *f(V)* = 0.217]. Ratings of perceived exertion were significantly higher during the mental fatigue condition compared to the control condition for 9 out of all subjects (**Figure 4B**).

#### Subjective workload

Following the whole-body ET (**Figure 2B**), none of NASA-TLX subscales presented any significant difference between conditions (all *p* > 0.050).

### EFFECTS OF MENTAL FATIGUE AND WHOLE-BODY ENDURANCE TASK ON NEUROMUSCULAR FUNCTION

#### Maximal voluntary contraction

**FIGURE 5:**
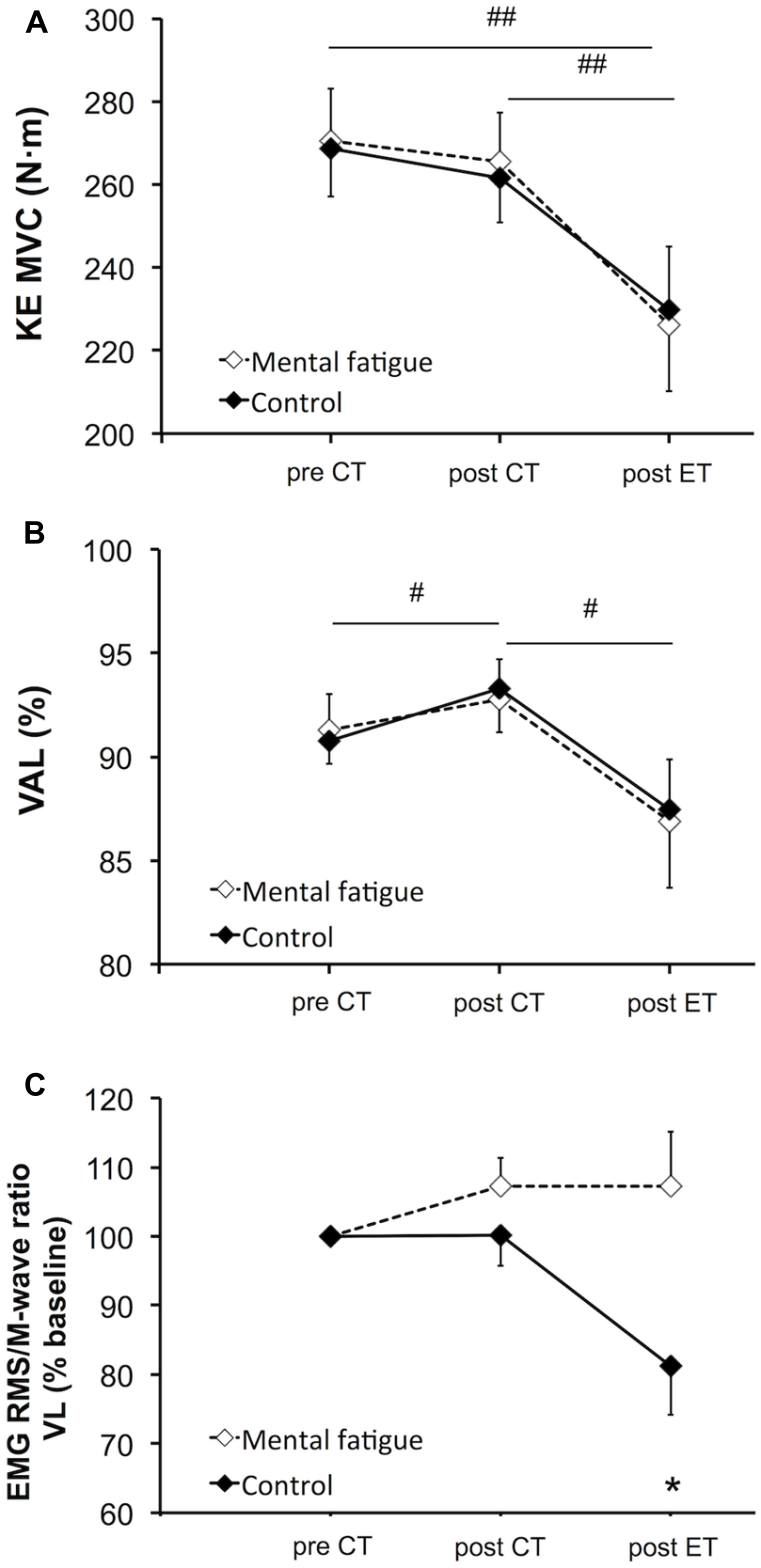
**Effects of mental fatigue on maximal voluntary contraction (MVC) of the knee extensors and central parameters of neuromuscular function.** MVC torque of the knee extensors (KE, **A**). Maximal voluntary activation level (VAL, **B**). RMS/Mmax (M-wave) ratio of the VL muscle **(C)**. CT, cognitive tasks; ET, whole-body endurance task; baseline, pre CT. # Significant main effect of time (*p* < 0.05). ## Significant main effect of time (*p* < 0.01). * Significant difference between conditions for the same time (*p* < 0.05). Data are presented as mean ± SEM.

There was no significant main effect of condition [*p* = 0.920, *f(V)* = 0.032] nor interaction [*p* = 0.515, *f(V)* = 0.204] on MVC torque of the knee extensors (**Figure 5A**). Follow-up tests on the significant main effect of time [*p* = 0.001, *f(V)* = 1.319] revealed that the CTs did not affect MVC torque [*p* = 0.194, *d*_z_ = 0.580]. The whole-body ET caused a significant reduction in MVC torque in both conditions (mental fatigue condition –17 ± 15%, control condition –15 ± 11%, *p* = 0.001, *d*_z_ = 1.890).

#### Peripheral fatigue

Peripheral parameters of neuromuscular function are presented in **Table 1**. There were no significant main effects of condition or interactions on all twitch parameters (all *p* > 0.050). Tw [*p* < 0.001, *f(V)* = 2.610], doublet [*p* < 0.001, *f(V)* = 1.636], Ct [*p* = 0.010, *f(V)* = 0.936], and RFD [*p* = 0.003, *f(V)* = 0.938] decreased significantly over time. The follow-up tests of the significant main effect of time are presented **Table 1**. M-wave amplitude of VL [*p* = 0.338, *f(V)* = 0.303] and RF [*p* = 0.079, *f(V)* = 0.584] muscles were not significantly affected by the CTs and the whole-body ET. M-wave amplitude of VL and RF muscles did not differ between conditions [*p* = 0.958, *f(V)* = 0.032 and *p* = 0.367, *f(V)* = 0.283] and did not show any interaction [*p* = 0.620, *f(V)* = 0.153 and *p* = 0.771, *f(V)* = 0.090].

**Table 1:** Effects of mental fatigue on peripheral parameters of neuromuscular function.

	Mental fatigue			Control		
	Pre CT	Post CT	Post ET	Pre CT	Post CT	Post ET
M-wave amplitude VL (mV)	17.77 ± 4.06	17.41 ± 3.99	18.35 ± 5.28	17.85 ± 3.85	17.69 ± 3.24	17.83 ± 3.97
M-wave amplitude RF (mV)	9.61 ± 2.59	9.17 ± 2.32	8.67 ± 2.49	9.13 ± 2.41	8.59 ± 2.10	8.37 ± 2.36
Tw (N.m)	60 ± 14	58 ± 15^££^	40 ± 12^£££§§§^	56 ± 15	54 ± 12^££^	36 ± 11^£££§§§^
Ct (ms)	76 ± 14	76 ± 10	67 ± 10^§^	79 ± 10	80 ± 1.09	69 ± 11^§^
RFD (N.m/s)	817 ± 243	780 ± 244	610 ± 219^££§^	727 ± 244	686 ± 192	526 ± 143^££§^
HRT (ms)	79 ± 27	83 ± 26	75 ± 27	78 ± 27	77 ± 29	72 ± 27
Doublet (N.m)	108 ± 16	105 ± 17^£^	87 ± 18^£££§§^	104 ± 19	95 ± 19^£^	83 ± 15^£££§§^

Ct, contraction time of the twitch; Tw, peak twitch; RFD, average rate of force development the twitch; HRT, half relaxation time of the twitch; CT, cognitive task; ET, whole-body endurance task; VL, vastus lateralis muscle; RF, rectus femoris muscle. ^£^ Main effect of time, significantly different from pre CT; ^§^ Main effect of time, significantly different from post CT. One item corresponds to *p* < 0.05, two items correspond to *p* < 0.01, and three items corresponds to *p* < 0.001. Data are presented as mean ± SD.

#### Central fatigue

Central parameters of neuromuscular function are presented in **Figure 5**. There was no significant main effect of condition [*p* = 0.869, *f(V)* = 0.054] or interaction [*p* = 0.672, *f(V)* = 0.201] on VAL (**Figure 5B**). Follow-up tests of the significant main effect of time [*p* = 0.011, *f(V)* = 0.990] revealed an increase in VAL post-CTs (*p* = 0.024, *d*_z_ = 0.438). On the contrary, the whole-body ET significantly reduced VAL (*p* = 0.013, *d*_z_ = 0.880). RMS/M-wave ratio of the VL muscle (**Figure 5C**) did not present any significant main effect of time [*p* = 0.313, *f(V)* = 0.318] or condition [*p* = 0.279, *f(V)* = 0.343]. Follow-up tests of the interaction [*p* = 0.021, *f(V)* = 0.810] revealed that the RMS/M-wave ratio of the VL muscle decreased only during the control condition following the whole-body ET (*p* = 0.038, *d*_z_ = 0.305). RMS/M-wave ratio of the RF muscle did not change overtime [*p* = 0.063, *f(V)* = 0.280] and did not present any main effect of condition [*p* = 0.915, *f(V)* = 0.032] or interaction [*p* = 0.335, *f(V)* = 0.335].

## DISCUSSION

The primary aim of this study was to test the hypothesis that mental fatigue exacerbates central fatigue induced by whole-body endurance exercise. The results of the present study do not support this hypothesis. Furthermore, mental fatigue did not exacerbate peripheral fatigue induced by whole-body exercise. Therefore, the higher-than-normal perception of effort experienced by mentally fatigued subjects is independent of any central or peripheral alteration of neuromuscular function.

### SELF-REGULATION, MENTAL FATIGUE, AND PERCEPTION OF EFFORT

We used a self-regulation task (incongruent Stroop task) to induce mental fatigue. The higher HR experienced during the incongruent Stroop task confirms that self-regulation is cognitively demanding and requires higher effort mobilization compared to the control task (Richter et al., 2008). The more demanding nature of the self-regulation task is also supported by higher ratings of mental demand, temporal demand, and effort compared to the control task. Moreover, the subjects presented a longer reaction time during the self-regulation task compared to the control task, confirming the presence of an additional cognitive control mechanism during the self-regulation task. As both control and self-regulation tasks involved sustained attention, the longer reaction time is likely to be due to the presence of response inhibition during the self-regulation task (Stroop, 1992; Sugg and McDonald, 1994).

Interestingly, both self-regulation and control tasks induced an increase in self-reported fatigue and a decrease in vigor suggesting presence of mental fatigue following both CTs. As in a previous study (Pageaux et al., 2014) a higher level of mental fatigue in the self-regulation condition was more clearly identified by higher RPE during the subsequent whole-body ET. However, it has to be noticed that perception of effort did not increase in the self-regulatory condition in three out of 12 subjects. This may be due to the fact that the self-regulation task was performed for only 30 min, and that this duration might be insufficient to induce mental fatigue in some subjects. Random day-to-day variability in perception of effort may also mask the effect of the self-regulation task at an individual level.

### MENTAL FATIGUE DOES NOT IMPAIR NEUROMUSCULAR FUNCTION

To check that mental fatigue did not alter neuromuscular function at the onset of the whole-body ET, we performed neuromuscular function tests before and after the CTs. According to previous studies, completion of short (Bray et al., 2008) or prolonged (Pageaux et al., 2013) CTs requiring self-regulation does not alter MVC of the handgrip and knee extensor muscles. Furthermore, another previous study (Rozand et al., 2014a) found that 80 intermittent maximal imagined contractions of the elbow flexor muscles did not alter MVC despite presence of mental fatigue. Our results are in line with these findings. Indeed, in our study, none of the CTs induced a significant decrease in knee extensors MVC.

Interestingly, as previously observed (Bishop, 2003), the absence of warm-up after the CTs impaired some peripheral parameters of neuromuscular function despite no reduction in knee extensors MVC. The absence of MVC torque reduction despite impaired muscle contractile properties can be explained by the slight increase in maximal voluntary activation of the knee extensor muscles measured post-CTs in both conditions. Indeed, an increase in VAL measured by the twitch-interpolated technique is likely to reflect an increase in muscle recruitment (Gandevia et al., 2013). Therefore, it is likely that our subjects compensated the absence of warm-up by slightly increasing muscle fibers recruitment and, thus, producing the same knee extensors MVC as prior to the CTs.

It has been suggested that CTs requiring self-regulation may cause the depletion of CNS resources, leading to reduced capacity of the CNS to recruit the working muscles (Bray et al., 2008, 2012). As both CTs did not induce a decrease in maximal muscle activation, our results and those of previous studies (Bray et al., 2008; Pageaux et al., 2013) do not support this hypothesis. However, because our study did not involve repeated MVCs, further studies are required to investigate the effect of mental exertion on the CNS capacity to recruit the working muscles during repeated contractions. The existing literature is not clear in this respect as both reduced MVC force (Bray et al., 2012) and no reductions in MVC torque and VAL (Rozand et al., 2014b) have been reported in experiments combining self-regulation tasks with repeated MVCs.

### MENTAL FATIGUE DOES NOT EXACERBATE CENTRAL FATIGUE INDUCED BY WHOLE-BODY ENDURANCE EXERCISE

Muscle fatigue can be caused by peripheral and/or central alterations (for review see Gandevia, 2001). As expected, mental fatigue did not exacerbate peripheral fatigue induced by the whole-body ET. The main aim of this study was to investigate whether mental fatigue exacerbates central fatigue induced by whole-body endurance exercise. Contrary to our hypothesis, the reduction in VAL induced by the whole-body ET did not differ between conditions. These results demonstrate for the first time that prolonged engagement with a CT requiring self-regulation does not exacerbate central fatigue during subsequent whole-body endurance exercise. The present findings are similar to those of our previous study showing that mental fatigue does not exacerbate central fatigue induced by submaximal single-joint exercise when measured at exhaustion (Pageaux et al., 2013). Therefore, the present study provides further evidence that the negative effect of mental fatigue on whole-body endurance performance (Marcora et al., 2009; MacMahon et al., 2014; Pageaux et al., 2014) is not mediated by central fatigue.

As mental fatigue does not affect the capacity of the CNS to recruit the working muscles (Pageaux et al., 2013; Rozand et al., 2014b), it is now clear that mental fatigue and central fatigue are two distinct phenomena. The most plausible explanation for the lack of interaction between mental fatigue and central fatigue is that these CNS functions involve different brain areas (Pageaux et al., 2013). Indeed, functional magnetic resonance imaging studies showed that central fatigue during index finger abduction exercise is associated with decrease in activation of the supplementary motor area and to a lesser extent, in parts of the paracentral gyrus, right putamen and in a small cluster of the left parietal operculum (van Duinen et al., 2007). Interestingly, none of these brain areas is significantly associated with CTs involving response inhibition. This cognitive control mechanism is significantly associated with activity of the pre-supplementary motor area and the anterior cingulate cortex (ACC; Mostofsky and Simmonds, 2008).

### MENTAL FATIGUE AND PHYSIOLOGICAL RESPONSES TO THE WHOLE-BODY ENDURANCE TASK

It has been shown previously that mental fatigue does not alter the cardiovascular, respiratory and metabolic responses to whole-body endurance exercise (Marcora et al., 2009). Our finding that the HR response to whole-body endurance exercise did not differ between conditions confirms this. Interestingly, however, the EMG RMS of the VL muscle during the whole-body ET was significantly higher following the self-regulation task compared to the control task. As cadence did not differ between conditions, this result suggests that prolonged self-regulation induced alterations in muscle recruitment at the onset and throughout the subsequent whole-body ET. This is not the first report of higher EMG amplitude during a physical task following a self-regulation task. In accordance with our results, Bray et al. (2008) measured higher EMG amplitude during sustained handgrip exercise following a short (3 min 40 s) engagement with the same incongruent Stroop task used in the present study. Therefore, our results, combined with those of Bray et al. (2008), suggest that both prolonged and short engagement with CTs requiring self-regulation can alter muscle recruitment during a subsequent physical task.

Because central and peripheral fatigue did not differ between conditions, higher EMG RMS of the VL muscle during the whole-body ET in the self-regulation condition cannot represent a compensatory increase in muscle recruitment. A possible explanation is that this EMG alteration represents an alteration in motor control in conditions of mental fatigue. This conclusion is supported by the findings of two recent studies showing that mental fatigue reduces mechanically induced tremor (Budini et al., 2014) and has adverse effects in all the three phases of slips (Lew and Qu, 2014). As injury in sport is more likely to occur in the late stage of an event or a season (e.g., Ekstrand et al., 2011), it seems that the effects of mental fatigue on motor control during whole-body physical tasks warrant further investigations.

### MENTAL FATIGUE AND PERCEPTION OF EFFORT

The higher-than normal perception of effort experienced by mentally fatigued subjects in the present experiment is similar to that reported in previous studies involving submaximal single-joint exercise (Pageaux et al., 2013) and whole-body endurance exercise (Marcora et al., 2009) at a fixed workload, as well as self-paced whole-body endurance exercise (Brownsberger et al., 2013; MacMahon et al., 2014; Pageaux et al., 2014). In some of these studies, the abnormal perception of effort has been associated with the negative effect of mental fatigue on endurance performance. However, despite strong evidences that mental fatigue increases RPE and impairs performance during endurance exercise, the underlying mechanisms of this alteration in perception of effort remain unclear.

It is well-accepted that, like any other perceptions, perception of effort results from the neurocognitive processing of sensory signals. However, the nature of the sensory signals involved in perception of effort generation remains debated. Briefly, two different theoretical models suggest that perception of effort reflects the neurocognitive processing of (i) signals from premotor/motor to sensory areas of the cortex during voluntary muscle contractions (corollary discharge model; Marcora, 2009; de Morree et al., 2012, 2014); or (ii) afferent sensory signals about the physiological condition of the body (interoception) and the environment (afferent feedback model; Hampson et al., 2001). Interestingly, in our study, mentally fatigued subjects experienced a higher-than-normal perception of effort despite no significant effects of mental fatigue on HR and peripheral fatigue. Because sensory signals from the heart and peripheral muscles are considered primary sources of afferent feedback for the generation of perception of effort (Hampson et al., 2001), it is unlikely that the higher RPE observed in our study reflects an alteration of afferent feedback induced by mental fatigue. Another possibility is that the higher than-normal perception of effort observed in mentally fatigued subjects reflects higher activity of premotor and/or motor areas of the cortex (i.e., higher central motor command) during whole-body endurance exercise. Although no direct neurophysiological measures of central motor command were taken in the present study, the abnormal EMG RMS of the VL muscle during the whole-body ET suggests that alterations in motor control may force mentally fatigued subjects to increase their central motor command in order to produce the same power output even when central and peripheral fatigue are not exacerbated. Finally, preliminary evidence that prolonged and demanding cognitive activity disrupts sensorimotor gating (van der Linden et al., 2006) suggests that mental fatigue may also affect the neurocognitive processing of the sensory signals underlying perception of effort. Further studies are required to investigate whether mental fatigue (i) alters the neurocognitive processing of the corollary discharges associated with central motor command, (ii) alters the central motor command itself, or (iii) alters the neurocognitive processing of afferent sensory signals.

Despite that we did not measure intrinsic changes in the brain induced by prolonged self-regulation leading to mental fatigue, it is possible to speculate on the mechanisms involved based on previous studies. The ACC is strongly activated during incongruent Stroop tasks (Bush et al., 1998; Swick and Jovanovic, 2002) and is also known to be linked with perception of effort (Williamson et al., 2001, 2002) and effort-based decision-making (Walton et al., 2006). Furthermore, studies with caffeine suggest an association between brain adenosine and mental fatigue (Lorist and Tops, 2003). It is therefore plausible that the higher perception of effort experienced by mentally fatigued subjects is caused by an accumulation of adenosine in the ACC. Indeed, experimental evidences that neural activity increases extracellular concentration of adenosine (Lovatt et al., 2012) and that brain adenosine accumulation reduces endurance performance (Davis et al., 2003) support this hypothesis. Further studies are required to confirm these speculations, and to investigate other cortical areas and neurotransmitters involved in the negative effects of mental fatigue on perception of effort and endurance performance.

## CONCLUSION

This study was the first to test the hypothesis that mental fatigue and central fatigue induced by whole-body exercise are causally related. Contrary to this hypothesis, our findings show that mental fatigue does not exacerbate central fatigue during subsequent whole-body exercise. However, we must acknowledge some limitations. Firstly, the whole-body ET had to be performed on a cycle ergometer, inducing a time delay between the end of exercise and the start of neuromuscular testing due to the need to transfer the participant from the cycle ergometer to the isokinetic dynamometer. Therefore, the extent of muscle fatigue is likely to be underestimated in both experimental conditions. Secondly, the whole-body ET consisted of 6 min of high-intensity cycling exercise at a fixed workload. Future studies should investigate the effects of mental fatigue on more prolonged low-to-moderate intensity whole-body endurance exercise including running where the extent of central fatigue may be greater (Millet and Lepers, 2004). The effects of mental fatigue on central fatigue induced by self-paced whole-body endurance exercise and repeated sprints also warrant further investigations given their relevance to both endurance competitions and team sports. Finally, brain activity during exercise was not measured in the present study and we can only speculate, based on previous studies, on the mechanisms underlying the increase in RPE observed in mentally fatigued subjects.

Despite these limitations, this study provides further evidences that mental fatigue does not reduce the capacity of the CNS to recruit the working muscles. Our results suggest that the negative effect of mental fatigue on perception of effort does not reflect a greater development of either central or peripheral fatigue. Consequently, mentally fatigued subjects are still able to perform maximal exercise, but they are experiencing an altered performance during submaximal exercise due to higher-than-normal perception of effort. Therefore, further studies should investigate the brain alterations underlying the negative effect of mental fatigue on perception of effort and endurance performance. A better understanding of these brain alterations could lead to development of novel targeted interventions to decrease perception of effort and improve endurance performance in athletes, and reduced exertional fatigue in patients (Macdonald et al., 2012).

## Conflict of Interest Statement

The authors declare that the research was conducted in the absence of any commercial or financial relationships that could be construed as a potential conflict of interest.
